# Screening for Atrial Fibrillation by Digital Health Technology in Older People in Homecare Settings: A Feasibility Trial

**DOI:** 10.1155/2024/4080415

**Published:** 2024-03-25

**Authors:** Edvard Liljedahl Sandberg, Sigrun Halvorsen, Trygve Berge, Jostein Grimsmo, Dan Atar, Bjørnar Leangen Grenne, Jarle Jortveit

**Affiliations:** ^1^Sorlandet Hospital, Department of Cardiology, Arendal, Norway; ^2^Institute of Clinical Medicine, University of Oslo, Oslo, Norway; ^3^Department of Cardiology, Oslo University Hospital Ullevaal, Oslo, Norway; ^4^Vestre Viken Hospital Trust, Baerum Hospital, Department of Medical Research and Department of Internal Medicine, Rud, Norway; ^5^Lovisenberg Rehabilitation, Cathinka Guldbergs Hospital, Department of Cardiac Rehabilitation, Oslo, Norway; ^6^LHL (National Organization for Heart and Lung Diseases), Jessheim, Norway; ^7^Clinic of Cardiology, St. Olavs Hospital, Trondheim, Norway; ^8^Department of Circulation and Medical Imaging, Norwegian University of Science and Technology, Trondheim, Norway

## Abstract

**Aims:**

Users of homecare services are often excluded from clinical trials due to advanced age, multimorbidity, and frailty. Atrial fibrillation (AF) is a common and frequently undiagnosed arrhythmia in the elderly and is associated with severe mortality, morbidity, and healthcare costs. Timely identification prevents associated complications through evidence-based treatment. This study is aimed at assessing the feasibility of AF screening using new digital health technology in older people in a homecare setting.

**Methods:**

Users of homecare services ≥ 65 years old with at least one additional risk factor for stroke in two Norwegian municipalities were assessed for study participation by nurses. Participants performed a continuous prolonged ECG recording using a patch ECG device (ECG247 Smart Heart Sensor).

**Results:**

A total of 144 individuals were assessed for study participation, but only 18 (13%) were included. The main reasons for noninclusion were known AF and/or anticoagulation therapy (25%), severe cognitive impairment (26%), and lack of willingness to participate (36%). The mean age of participants performing the ECG test was 81 (SD ± 7) years, and 9 (50%) were women. All ECG tests were interpretable; the mean ECG monitoring time was 104 hours (IQR 34-338 hours). AF was detected in one individual (6%).

**Conclusion:**

This feasibility study highlights the challenges of enrolling older people receiving homecare services in clinical trials. However, all included participants performed an interpretable and prolonged continuous ECG recording with a digital ECG patch device. This trial is registered with NCT04700865.

## 1. Introduction

Older people are often excluded from clinical trials due to advanced age, multimorbidity, and/or frailty [1]. Recruitment and retention of older participants are important challenges. Physical attendance and personal requests may be needed for successful recruitment. Cognitive decline is common, and proxies or next-to-kin are often needed to consent. Older participants have an increased risk of dropping out of trials, and several other pitfalls may result in both underestimating and overestimating the intervention effects [1]. Despite these obstacles, it is important to include elderly individuals in clinical trials. Reliable evidence is needed for the successful treatment, management, and care of older people.

Atrial fibrillation (AF) is a common heart rhythm disorder, especially in the elderly [2–6]. AF may be undiagnosed due to its paroxysmal and often asymptomatic nature [7, 8]. The risk of AF-associated complications includes stroke, heart failure, dementia, depression, and reduced quality of life, and the prevalence increases with advancing age [8]. The risk of stroke can be significantly reduced in patients with AF by oral anticoagulation therapy; both the potential for risk reduction as well as the risk of its adverse effects are higher in older people [8, 9]. Screening for AF is recommended in people > 75 years of age and individuals at an increased risk of stroke [8]. However, the oldest and most frail patients, i.e., those with the greatest potential to benefit from the detection and treatment of AF, have not been included in all AF screening trials.

This study is aimed at assessing the feasibility of AF screening using a new and simple patch ECG device in older people in a homecare setting.

## 2. Methods

### 2.1. Study Design

This prospective open-label nonrandomized feasibility study was conducted and reported according to the “CONSORT 2010 statement: extension to randomized pilot and feasibility trials” [10]. Sorlandet Hospital, Arendal, Norway, was the responsible study centre.

### 2.2. Study Population

All users of the homecare services in two small Norwegian municipalities (Birkenes and Lillesand, total population of approximately 16900 inhabitants [11, 12]) were assessed for study participation by nurses in the homecare services between the 1^st^ of October 2022 and the 28^th^ of February 2023. Individuals fulfilling the inclusion and having no exclusion criteria were included after signing consent for participation.

#### 2.2.1. Homecare Services

In Norway, the homecare service provides medical, nursing, therapeutic, and/or other support to individuals, mostly older people, living in private homes. The homecare service aims to assist people recovering from an illness, managing a chronic condition, or dealing with disabilities. Municipal health services operate homecare services and are primarily staffed by nurses collaborating closely with general practitioners (GPs).

#### 2.2.2. Inclusion Criteria

The study had the following inclusion criteria: user of the homecare services, age ≥ 65 years with minimum one other risk factor for stroke according to the CHA_2_DS_2_-VASc risk score [8] (age ≥ 75 years, female gender, diabetes, heart failure, hypertension, previous stroke/TIA, and/or vascular disease), and willingness to participate in the study. No prior experience in handling technological tools was necessary as they received personal assistance from the homecare staff.

#### 2.2.3. Exclusion Criteria

Individuals with a prior diagnosis of AF, currently receiving anticoagulation therapy, cognitive impairment incompatible with informed consent, and/or the ability to cooperate about the study procedure were excluded from participation in the study.

### 2.3. Digital Health Technology

The screening procedures were performed with the ECG247™ Smart Heart Sensor system (Appsens AS, Lillesand, Norway, http://www.ecg247.com). The system consists of a disposable ECG electrode patch, a reusable sensor, and a medical-grade smartphone application with immediate transfer of ECG recordings to a secure medical back-end cloud service with real-time ECG analysis [13]. ECG247 Smart Heart Sensor is designed according to the General Data Protection Regulation (GDPR) requirements and is CE-certified according to the EU Medical Device Directive (93/42/EEC). The system has improved diagnostic accuracy and usability compared to conventional Holter technology and allows for high ECG quality even during physical activity [14, 15]. ECG247 Smart Heart Sensor has recently demonstrated excellent feasibility for AF screening in the general population ≥ 65 years of age [16]. The equipment cost per test was approximately 35 euro.

### 2.4. Study Procedure

Three nurses in the homecare service in each municipality were trained in the study procedure.

All participants reported their medical history regarding stroke, transient ischemic attack, systemic embolism, heart failure, hypertension, diabetes mellitus, myocardial infarction, coronary revascularization, and other vascular diseases in a study-specific questionnaire. Data on height, weight, and medication were also collected. The process was assisted by the homecare service staff. A web-based solution from Services for Sensitive Data, University of Oslo, Norway, was used.

**Figure 1:**
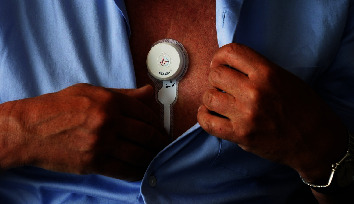
Digital health technology (ECG247 Smart Heart Sensor).

The homecare service staff assisted the study participants in downloading the ECG247 app and adhere the patch ECG device over the sternum (Figure 1). Participants who did not own a smartphone were offered to borrow a study phone. A minimum 24-hour test period was recommended, but everyone was encouraged to continue the recording for as long as possible.

**Figure 2:**
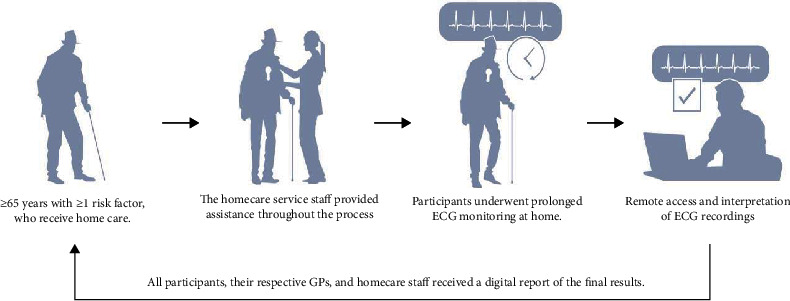
Study procedure.

All ECG recordings were reviewed remotely by a trained and experienced cardiology fellow (ELS) at Sorlandet Hospital Arendal (Figure 2). The following variables were registered: duration of the heart rhythm recording, heart rhythm (sinus rhythm, atrial fibrillation/flutter (AF) > 30 sec, supraventricular tachycardia (SVT) > 15 sec, ventricular tachycardia (VT) (>4 beats), and pause (≥4 sec)), duration, and heart rate of arrhythmias. A cardiologist confirmed all abnormal ECGs and all arrhythmias.

All participants received a digital report of the study results.

### 2.5. Outcomes

The primary outcome was the feasibility of AF screening by digital health technology in older people in a homecare setting, i.e., the proportion of potential participants who were included and completed an interpretable long-term ECG recording with the ECG247 Smart Heart Sensor. The study involved no follow-up after completion of the ECG test.

### 2.6. Statistics

Continuous variables are presented as mean ± SD (standard deviation) or median (25^th^ and 75^th^ percentile). Categorical variables are presented as numbers and percentages. Proportions are given by nonmissing values. The analyses were performed using STATA, version 17 (StataCorp, College Station, TX, USA).

### 2.7. Patient and Public Involvement

A user representative was consulted in the preparation of the study protocol, and feedback from participants was used to adjust the study procedure within the protocol.

### 2.8. Ethics

The study was approved by the Regional Committee for Medical and Health Research Ethics (REK 408516). All participants signed informed consent for study participation.

## 3. Results

**Figure 3:**
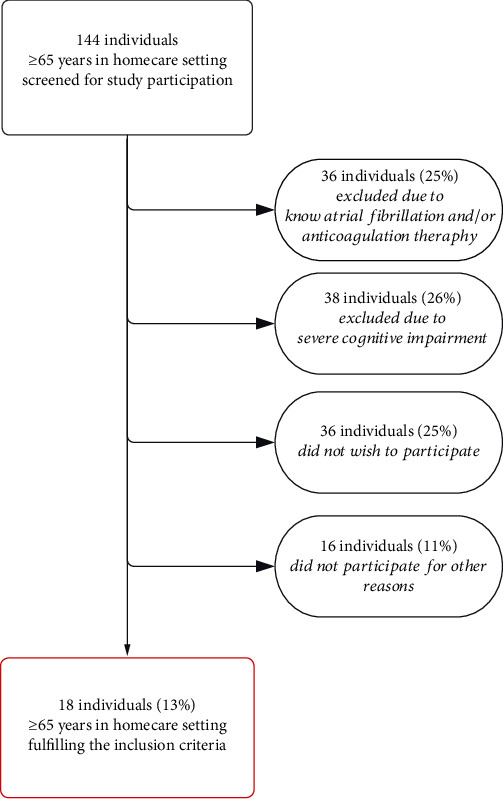
Flowchart (assessed vs. included participants).

A total of 144 people ≥ 65 years in homecare settings were assessed for study participation from October 1, 2022, to February 28, 2023. The majority did not meet the inclusion criteria or had exclusion criteria (Figure 3). Only 18 (13%) of the 144 assessed individuals were included in the study and underwent long-term ECG monitoring.

The clinical characteristics of the study population are presented in Table 1. The mean age was 81 (SD ± 7) years, and nine (50%) were women.

The mean ECG monitoring time was 104 hours (IQR 34–338 hours). In 14 (78%) participants, the ECG monitoring time was >72 hours. All ECG recordings were interpretable. AF was detected in one individual (6%). In this individual, the total recording spanned 111 hours, with the onset of AF after 10 hours and lasting for 10 hours. No other significant arrhythmias were detected in the study.

## 4. Discussion

This feasibility study highlights the challenges of recruiting older people in homecare settings in clinical trials. Of the 144 individuals assessed, only 18 (13%) were included in the study. Frequent reasons for nonparticipation were the presence of AF and the current use of oral anticoagulation (OAC) therapy. Cognitive impairment and other comorbidities also influenced exclusion from the study. Noteworthy, 36% declined participation for various reasons. However, all the included participants performed an interpretable and prolonged continuous ECG procedure by new digital health technology (ECG247 Smart Heart Sensor). Previously undetected AF was found in one (6%) individual.

Despite updated guidance for clinical trials in the geriatric population, elderly people are still underrepresented in such studies [17, 18]. Older people may respond differently than younger patients to tests and treatments, and the exclusion of older patients with multimorbidity and polypharmacy challenges the general validity of many clinical trials [19]. This important limitation must be considered when applying findings of clinical trials in old individuals.

To our knowledge, this study represents the first remote AF screening trial utilizing new digital health technology among individuals receiving homecare services in Norway. One of the most important findings was the low inclusion rate, which can be attributed to various factors. As mentioned, a frequent reason for noninclusion was the presence of a prior diagnosis of AF and/or the use of anticoagulation therapy. Furthermore, individuals with severe cognitive impairment were excluded due to challenges related to obtaining informed consent and their ability to cooperate with the study procedure. Varying degrees of dementia are prevalent among users of homecare services, and routine measures exist for obtaining informed consent from their closest proxies. However, this is time-consuming and resource-intensive, posing challenges for busy homecare services to prioritize. Nonetheless, these distinctions are important, as a prior diagnosis of AF and the current use of OAC directly impact AF screening. Conversely, cognitive impairment and reduced willingness to participate pertain to generally challenging the enrolment of older people in clinical research.

Despite the dedicated commitment and support from the homecare service staff, the nonparticipation of 36% of individuals due to unwillingness for various reasons is notable and has implications, particularly since the context was in a homecare setting. It raises concerns regarding the generalizability and representativeness of the study findings. Unfortunately, we did not obtain a comprehensive explanation for their unwillingness, leaving us to only speculate about the underlying reasons. In general, older people's willingness and engagement with digital health technology depend on factors such as previous experience, perceptions of personal benefit, digital skills, the usability of the technology, and support from healthcare professionals. Understanding these factors becomes important in developing targeted strategies that address the specific concerns and barriers that older individuals face.

Digital health technology encompasses a wide range of applications addressing different health needs. Digital health technology has the potential to revolutionize healthcare delivery and research by streamlining operations, improving patient outcomes, decreasing healthcare costs, and facilitating recruitment, randomization, data collection, and implementation [20]. Digital health technology has several applications in cardiovascular medicine, e.g., self-testing and self-management, remote monitoring, decision support, virtual communication, and education. The SARS-CoV-2 (COVID-19) pandemic accelerated the development of digital health technology, but the uptake beyond the telecare systems has been slow [21]. Important healthcare system barriers like concerns about costs, effectiveness, and safety are impeding the proposed digital health revolution [22].

AF fulfills most criteria for a screening program; it is a prevalent, often asymptomatic disease with available and effective treatment for preventing complications [23]. International guidelines recommend opportunistic screening for AF in older people at increased risk of stroke [8]. However, randomized controlled trial data to confirm the health benefits of screening for AF and inform the choice of optimal screening programs and strategies for implementation are scarce. While beyond the scope of the current study, the different results of the STROKESTOP and LOOP trials reinforce the need for further trials to assess the net efficacy of AF screening [24, 25]. We have recently demonstrated the feasibility of self-screening for AF by the ECG247 Smart Heart Sensor in the general population ≥ 65 years old with additional risk factors for stroke [16]. Despite the low inclusion rate, this study among elderly users of homecare services confirms the technical feasibility of performing AF screening in eligible individuals in this population.

AF often has a paroxysmal (intermittent) course and consequently may be missed by single ECG recordings [26, 27]. Conventional hospital-based Holter monitoring systems are cumbersome to use in homecare services due to high competence requirements and high operational costs. Several previous AF screening studies have used single time point or intermittent ECG for screening, which runs the risk of missing cases of paroxysmal AF [26]. Furthermore, older individuals with arthritic fingers *or movement disorders such as parkinsonism* may find it challenging to operate handheld devices [28, 29]. New digital health technology, like the ECG247 Smart Heart Sensor, enables prolonged continuous ECG monitoring at low cost and low personnel requirements.

This study has several important limitations. It was a feasibility study enrolling a limited number of participants in a limited geographical area and cannot estimate the prevalence of AF among users of homecare services. We did not collect data on time spent by the homecare service staff nor for the manual review of the ECG recordings, and accurate costs for the screening procedure cannot be estimated. Furthermore, we had no opportunity to validate the patient-reported health information of the study participants. Only one physician assessed the ECG recordings. The diagnostic accuracy of the device has been described previously, but some arrhythmia episodes might be undetected by the system [14].

## 5. Conclusion

In conclusion, it is challenging to enrol elderly users of homecare services into clinical trials utilizing digital health technology. However, AF screening by easy-to-use digital health technology is feasible in a selected and motivated geriatric population. We recommend including more older adults receiving homecare services in future AF screening trials.

## Figures and Tables

**Table 1:** Clinical characteristics of the study population, *n* = 18.

	*n*
Women (%)	9 (50)
Mean age (years) (SD)	81 (7)
Mean body mass index (kg/m^2^) (SD)	27 (6)
Smoking (%)	1 (8)
Hypertension (%)	10 (78)
Diabetes (%)	6 (46)
Previous coronary heart disease	
Myocardial infarction (%)	2 (15)
Percutaneous coronary intervention (%)	3 (23)
Coronary artery bypass grafting (%)	2 (15)
Previous stroke (%)	2 (15)
Peripheral artery disease (%)	0 (0)
Heart failure (%)	2 (15)
Hypothyroidism (%)	3 (23)
Hyperthyroidism (%)	0 (0)
Chronic obstructive pulmonary disease (%)	1 (8)
Sleep apnoea (%)	1 (8)
Mean CHA_2_DS_2_-VASc risk score (SD)	3.7 (1.4)
Median CHA_2_DS_2_-VASc risk score (IQR)	3 (3-4)
Medication use	
Acetylsalicylic acid (%)	9 (69)
Anticoagulation therapy (%)	0 (0)
Lipid-lowering therapy (%)	12 (92)
Beta blocker (%)	6 (46)
Angiotensin-converting enzyme (ACE) inhibitors (%)	3 (23)
Angiotensin II receptor blockers (%)	8 (62)

SD: standard deviation; IQR: interquartile range; CHA_2_DS_2_-VASc: congestive heart failure, hypertension, age ≥ 75 years (doubled), diabetes, stroke (doubled), vascular disease, age 65 to 74 years and sex category (female).

## Data Availability

The data underlying this article will be shared on reasonable request to the corresponding author (Edvard Liljedahl Sandberg).

## Additional Points

*What's New*. The majority of elderly people in a homecare setting were not eligible for inclusion or did not want to participate in a screening for atrial fibrillation trial. However, all included individuals performed the technical aspect of the patch ECG screening test.
